# Supplementary material for: The Effect of Enteral Immunonutrition in the Intensive Care Unit: Does It Impact on Outcomes?
Source: Nutrients. 2022 May 1;14(9):1904. doi: 10.3390/nu14091904 (PMC9103218; doi:10.3390/nu14091904)
Supplement: Supplementary file 1 [file nutrients-14-01904-s001.zip › nutrients-1677201-supplementary.pdf]

Analysis of the amount of delivered enteral nutrition (EN)

An excessive volume administered within the EN subgroup may have potentially altered our results. Patients who receive higher amount (i.e., volume) of delivered EN may have trend towards suffering from higher gastric residual volume. In consequence, nutrition would potentially have been stopped more frequently in those patients and those patients received lower amount of nutrition. We have largely calculated this parameter and we did not find any difference. As we underlined within the manuscript, the mean volume of delivered EN was similar between groups (845 ± 384mL in the EN subgroup *vs* 892 ± 342mL in the EN-INM subgroup; *P=0.85*) (see **Table S1**)

Table S1. Mean delivered volume of enteral nutrition during nutrition therapy.

**Day**	**Mean delivered volume of enteral nutrition**		
	**EN**	**EN-IMN**	***P***
**1**	348 ± 345	368 ± 225	*0.75*
**2**	537 ± 344	727 ± 284	*0.24*
**3**	815 ± 554	915 ± 354	*0.13*
**4**	990 ± 450	940 ± 360	*0.45*
**5**	870 ± 355	867 ± 413	*0.85*
**6**	895 ± 428	915 ± 398	*0.47*
**7**	910 ± 440	892 ± 423	*0.85*
**8**	946 ± 405	956 ± 398	*0.90*
**9**	962 ± 454	929 ± 445	*0.87*
**10**	990 ± 464	1011 ± 370	*0.35*
**11**	1008 ± 495	1058 ± 376	*0.86*
**12**	1022 ± 382	1172 ± 223	*0.20*
**13**	1045 ± 323	1090 ± 313	*0.92*
**14**	1083 ± 389	1162 ± 322	*0.78*

EN: Enteral nutrition formula; IMN: Immunonutrition formula.

It is important to remark that caloric and protein delivery not only depends on EN volume. Delivery may also depend on characteristics of the administered formula (e.g., calories per mL), the better tolerance of the formula based on the composition (e.g., type of protein), the clinical status of the patient (e.g., higher illness severity is related with lower tolerance). All those factors are difficult to evaluate and closely related with all types of research in nutrition therapy in critically ill patients.

Analysis of the ratio of delivered energy and protein /required energy and protein

The mean ratio of delivered/required energy (0.79 ± 0.6 in the EN subgroup *vs* 0.84 ± 0.5 in the EN-INM subgroup; *P=0.45*) and delivered/required protein (0.77 ± 0.6 in the EN subgroup *vs* 0.85 ± 0.6 in the EN-INM subgroup; *P=0.55*) although differed slightly in some time-points (see **Table S2**). Required energy and protein intakes were considered as those requirements calculated by the physician on charge to be delivered.

Please, note that the study was observational but patients received a progressive increase of the prescribed dosage during the initiation of EN until full prescribed dose 72h after the initiation of enteral nutrition. This progressive delivery may be intuitively showed in Figure 2. We remember that based on our current practice, we initially prescribed dosage about 25-35% on day 1, 60-70% on day 2, and full dose on day 3. Indeed, non-nutritional caloric requirements (e.g., dextrose serum) were considered for caloric calculation.

Table S2. Mean ratio of delivered/required energy and protein delivery during enteral nutrition therapy.

**Day**	**Mean ratio of delivered/required energy**			**Mean ratio of delivered/required protein**		
	**EN**	**EN-IMN**	***P***	**EN**	**EN-IMN**	***P***
**1**	0.85±0.05	0.88±0.06	*0.35*	0.65±0.07	0.82±0.07	***0.04***
**2**	0.75±0.11	0.84±0.04	*0.08*	0.70±0.14	0.85±0.09	*0.08*
**3**	0.65±0.14	0.85±0.08	***0.003***	0.64±0.14	0.86±0.05	***0.04***
**4**	0.75±0.05	0.82±0.04	***0.04***	0.66±0.11	0.84±0.08	***0.04***
**5**	0.80±0.09	0.82±0.06	*0.85*	0.74±0.09	0.82±0.05	*0.75*
**6**	0.74±0.06	0.80±0.04	*0.75*	0.76±0.10	0.80±0.06	*0.75*
**7**	0.72±0.04	0.79±0.03	*0.50*	0.74±0.11	0.80±0.08	*0.45*
**8**	0.74±0.06	0.82±0.03	*0.20*	0.76±0.12	0.83±0.09	*0.50*
**9**	0.79±0.04	0.85±0.04	*0.90*	0.82±0.08	0.86±0.05	*0.90*
**10**	0.82±0.03	0.86±0.03	*0.15*	0.81±0.06	0.85±0.05	*0.55*
**11**	0.83±0.04	0.84±0.04	*0.10*	0.84±0.05	0.86±0.07	*0.85*
**12**	0.82±0.03	0.87±0.03	***0.04***	0.82±0.03	0.87±0.06	*0.35*
**13**	0.83±0.03	0.88±0.03	*0.09*	0.85±0.06	0.87±0.05	*0.15*
**14**	0.82±0.03	0.88±0.04	*0.07*	0.82±0.04	0.86±0.06	*0.07*

EN: Enteral nutrition formula; IMN: Immunonutrition formula.

Analysis of the amount of delivered Carbohydrates & Lipids

As we pointed out in Discussion section, the trend towards a better caloric and protein delivery in the patients who received IMN formula may be related with the own composition of the formula.

The caloric delivery could be related also with other different components of the formula, such as carbohydrates and lipids, rather than proteins. We found similar mean carbohydrate delivery in the EN-INM subgroup (149±63.2 g·day^{-1} in the EN subgroup *vs* 152.4±58.9 g·day^{-1} in the EN-INM subgroup; *P=0.65*), except a slight trend towards better delivery during the first days of EN (see **Table S3**), whereas no difference was shown regarding the mean amount of delivered lipids (41.7±17.1 g·day^{-1} in the EN subgroup *vs* 43.8±17.1 g·day^{-1} in the EN-INM subgroup; *P=0.82*) (see **Table S4**).

We hypothesized that the trend towards improvement in caloric delivery in the EN-IMN subgroup is related with the additive caloric effect of all the separate components of the EN, especially protein component. Indeed, proteins reached statistical significance in the multivariate analysis (Tables 2 & 6).

Table S3. Mean delivered carbohydrates (g day^{-1}) of enteral nutrition during nutrition therapy.

Day	Mean delivered Carbohydrates (g · day^{-1}) during enteral nutrition		
	**EN**	**EN-IMN**	***P***
**1**	58.2 ± 55.2	63.6 ± 37.5	*0.65*
**2**	87.2 ± 43.4	124.3 ± 49.2	*0.14*
**3**	105.1 ± 45.4	155.3 ± 60.3	*0.10*
**4**	112.3 ± 56.8	162.6 ± 62.3	*0.25*
**5**	135.3 ± 45.6	151.1 ± 68.5	*0.45*
**6**	132.6 ± 78.5	158.3 ± 67.6	*0.47*
**7**	138.7 ± 68.7	155.3 ± 73.2	*0.55*
**8**	146.2 ± 75.4	165.4 ± 69.2	*0.72*
**9**	144.1 ± 75.9	161.2 ± 75.6	*0.81*
**10**	155.8 ± 66.7	176.5 ± 64.1	*0.78*
**11**	160.2 ± 65.4	183.1 ± 65.2	*0.77*
**12**	182.5 ± 63.9	202.7 ± 39.6	*0.70*
**13**	185.8 ± 63.5	188.6 ± 54.2	*0.90*
**14**	183.6 ± 61.2	201.1 ± 55.7	*0.82*

EN: Enteral nutrition formula; IMN: Immunonutrition formula.

Table S4. Mean delivered lipids (g day^{-1}) of enteral nutrition during nutrition therapy.

**Day**	**Mean delivered lipids (g · day^{-1}) of enteral nutrition**		
	**EN**	**EN-IMN**	***P***
**1**	16.5 ± 12.1	18.4 ± 11.3	*0.85*
**2**	34.7 ± 15.8	36.5 ± 14.2	*0.76*
**3**	42.2 ± 18.9	45.2 ± 17.9	*0.84*
**4**	42.7 ± 19.7	46.7 ± 17.7	*0.65*
**5**	43.5 ± 19.6	43.4 ± 20.6	*0.90*
**6**	43.9 ± 18.7	45.5 ± 19.7	*0.87*
**7**	42.3 ± 23.2	44.5 ± 21.2	*0.95*
**8**	44.5 ± 19.2	47.8 ± 19.8	*0.90*
**9**	45.9 ± 23.3	46.4 ± 22.5	*0.95*
**10**	46.8 ± 19.5	49.8 ± 18.5	*0.85*
**11**	49.9 ± 20.8	52.9 ± 18.8	*0.88*
**12**	56.4 ± 16.3	58.6 ± 11.2	*0.95*
**13**	52.6 ± 20.5	54.5 ± 15.6	*0.91*
**14**	52.8 ± 18.7	58.1 ± 15.9	*0.85*

EN: Enteral nutrition formula; IMN: Immunonutrition formula.
